# An anomalous addition of chlorosulfonyl isocyanate to a carbonyl group: the synthesis of ((3a*S*,7a*R*,*E*)-2-ethyl-3-oxo-2,3,3a,4,7,7a-hexahydro-1*H*-isoindol-1-ylidene)sulfamoyl chloride

**DOI:** 10.3762/bjoc.15.89

**Published:** 2019-04-16

**Authors:** Aytekin Köse, Aslı Ünal, Ertan Şahin, Uğur Bozkaya, Yunus Kara

**Affiliations:** 1 Department of Chemistry, Aksaray University, 68100 Aksaray, Turkey; 2 Department of Chemistry, Hacettepe University, 06800 Ankara, Turkey; 3 Department of Chemistry, Atatürk University, 25240 Erzurum, Turkey

**Keywords:** addition reaction, chlorosulfonyl isocyanate, sulfamoyl chloride, theoretical calculations

## Abstract

In this study, we developed a new addition reaction of chlorosulfonyl isocyanate (CSI), starting from 2-ethyl-3a,4,7,7a-tetrahydro-1*H*-isoindole-1,3(2*H*)-dione. The addition reaction of CSI with 2-ethyl-3a,4,7,7a-tetrahydro-1*H*-isoindole-1,3(2*H*)-dione resulted in the formation of ylidenesulfamoyl chloride, whose exact configuration was determined by X-ray crystal analysis. We explain the mechanism of product formation supported by theoretical calculations.

## Introduction

**Scheme 1:**
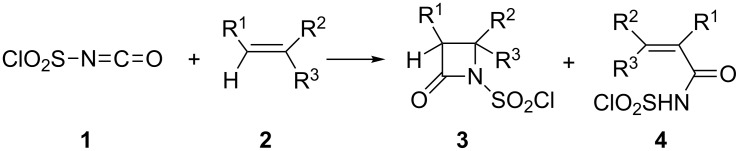
The reaction of CSI with olefins.

Since its identification in 1959 [1], chlorosulfonyl isocyanate (CSI, **1**) continues to be the most reactive isocyanate to date. CSI is relatively more reactive than alkylsulfonyl isocyanate in olefin additions [2]. Its highly reactive nature is due to the polarization of the allene double bond by the highly electronegative chlorosulfonyl group. CSI reacts with unsaturated systems to yield either *N*-chlorosulfonyl-β-lactams **3** or unsaturated *N*-chlorosulfonyl amides **4** (Scheme 1).

**Figure 1:**
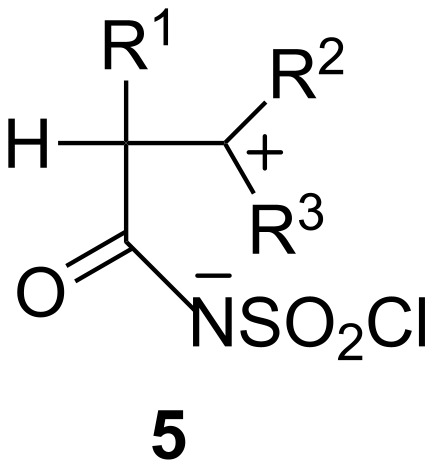
The dipolar intermediate formed in the reaction of CSI with olefins.

β-Lactams **3** generally predominate and in many cases are the exclusive products and they serve as key substances in a variety of chemical transformations. When chlorosulfonyl amides **4** are produced, they may be converted to other compounds*.* Graf [1] proposed that the reaction of CSI with unsaturated systems proceeds via the direct formation of dipolar intermediate **5** which may undergo a ring closure to form **3** or a hydrogen transfer to afford **4** (Figure 1).

A wide variety of reactions has been reported for CSI, among which its addition to unsaturated systems has proven to be the most interesting and synthetically useful [3]. The addition of CSI to unsaturated systems and its further reactions have been examined by various groups [4–11]. On the other hand, examples for the reaction of carbonyl group containing compounds with CSI are limited in the literature. In particular, compounds containing an amide or pyrone skeletal structure were used in these studies. These reactions were reported by the research groups of Reynolds [12], Sattur [13], Jiménez [14] and Schwarz [15]. In our continuing studies on the synthesis of isoindole derivatives, we have included the addition of CSI to unsaturated systems and obtained unexpected results. When the reactions were performed by heating without any solvent, the condensation product imine was the sole product. In this paper, we present for the first time a unique example describing the addition of CSI to a system comprising both independent double bonds and imide functional groups. The mechanism for the addition of CSI to a carbonyl group is explained by theoretical computations.

## Results and Discussion

**Scheme 2:**
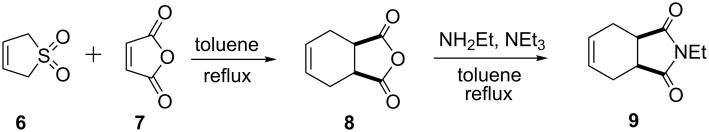
The synthesis of imide **9**.

Our starting material was 2-ethyl-3a,4,7,7a-tetrahydro-1*H*-isoindole-1,3(2*H*)-dione (**9**), which we have synthesized in previous studies [16–17]. Imide **9** was synthesized via the cycloaddition of 3-sulfone to maleic anhydride. The reaction of ethylamine with anhydride **8** in the presence of a toluene/triethylamine mixture (3:1) produced imide **9** in 80% yield (Scheme 2).

**Scheme 3:**
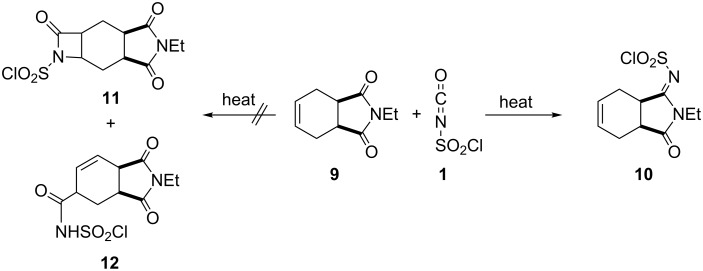
The synthesis of ylidene sulfamoyl chloride **10**.

To synthesize a new lactam derivative of isoindole-1,3-dione, we investigated the reaction of imide **9** with CSI in toluene at room temperature. Nevertheless, the starting imide **9** did not react with CSI, and we could not obtain the expected products lactam **11** and amide **12**. Next, we studied the reaction of **9** with CSI by heating without solvent, which produced a very interesting product **10** that contained a *N*-chlorosulfonylimine group (Scheme 3).

**Figure 2:**
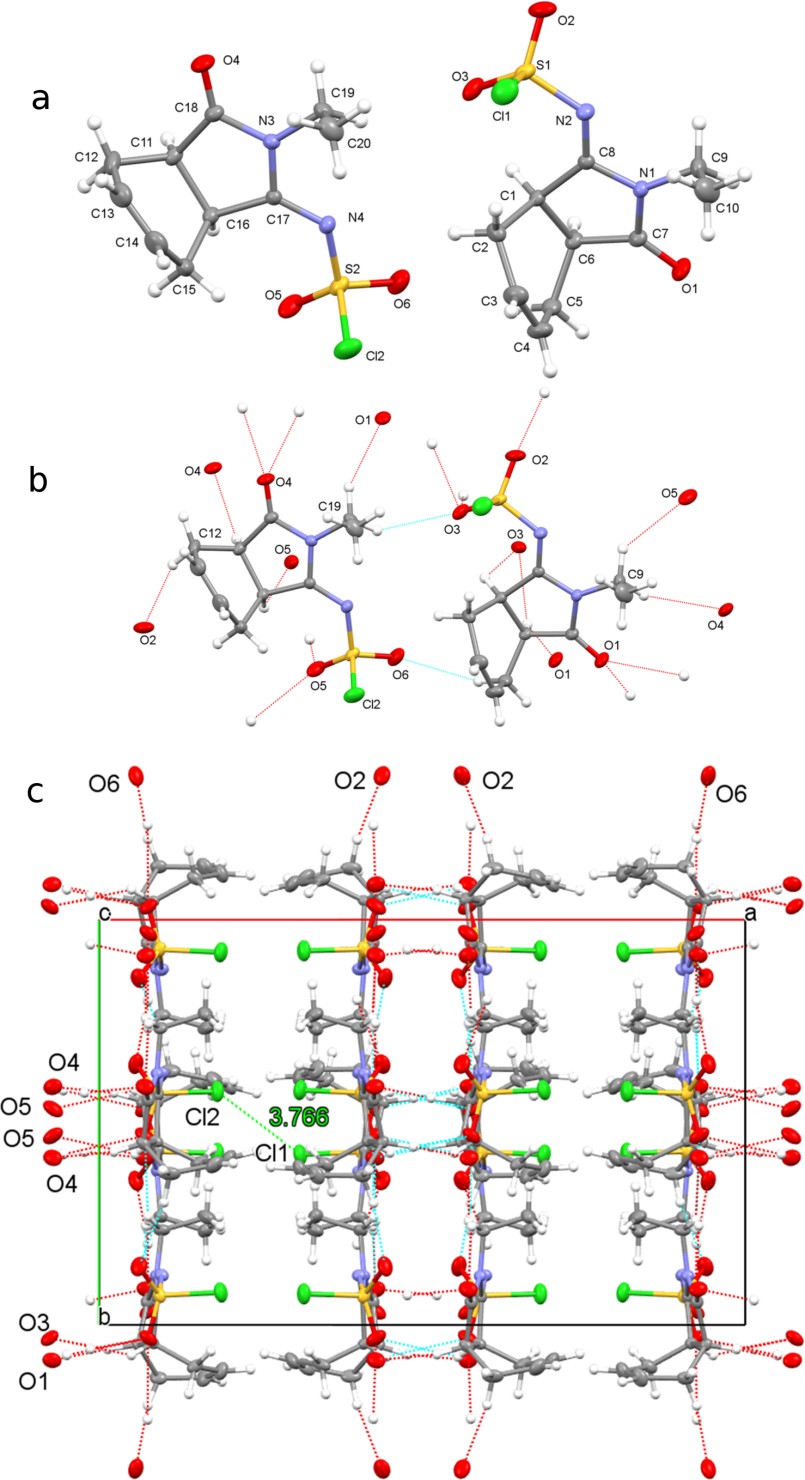
(a) Molecular structure of racemic molecule **10** (asymmetric unit). Thermal ellipsoids are drawn at the 30% probability level. (b) Geometric parameter with H-bonded geometry. Hydrogen bonds are drawn as dashed lines. (c) Stacking motif with the unit cell viewed downward along the c-axis. Dashed lines indicate C–H∙∙∙O interactions.

Upon examination, the most conspicuous features in the ^1^H and ^13^C NMR spectra of **10** were the change in the molecular symmetry and the chemical shifts of the carbonyl groups in the molecule. If the amide compound **12** had formed, there would be a change in the symmetry of the molecule, but the ^1^H and ^13^C NMR spectra do not support the structure of amide **12**. The results of double resonance experiments clearly indicated that the HC=CH double bond was located between two CH_2_ groups (C-4 and C-7), similar to those present in the starting compound. Additionally, there was no signal of the three different carbonyl carbons in the ^13^C NMR spectrum of **10**. On the other hand, the presence of double bond protons, three (CH_2_) groups, two CH protons, and one methyl (CH_3_) group, such as those in the starting compound, reveal that the reaction takes place on the carbonyl groups in the imide ring. While the ^1^H and ^13^C NMR spectra support a structure, we were not sure what kind of structure was indicated based on the spectral data. We determined the exact structure of **10** by X-ray crystal analysis (Figure 2) [18].

In addition to determining its structure, we performed X-ray crystal analysis of molecule **10** to identify the possible interactions. The structure has a racemic form, with the atoms of racemate **10** labeled and the polymeric *H*-bonding geometries shown in Figure 2a. We provide crystallographic data and structural refinement details in the experimental section. Compound **10** crystallizes in the monoclinic space group *C*2/*c*. The C–C (cyclohexene) distances are in the typical single bond range [1.491(3)–1.356(3) Å]. The C=C double bonds are in the cyclohexene units range between 1.307–1.316(3) Å and the S=O bonds are between 1.417–1.414(3) Å. The conformation is defined by steric effects, which force a fold of the cyclohexene rings relative to the mean plane through the pyrrolidine group. For both enantiomers, the cyclohexene ring has a twist-boat conformation and the pyrrolidine rings are in half-chair conformation. The structures contain four asymmetric carbon atoms and the stereogenic centers are as follows: C1(*R*), C6(*S*), C11(*R*), and C16(*S*), where the *N*-chlorosulfonyl group attached to the carbonyl atom changes the stoichiometry. In the solid state, compound **10** is stabilized via effective intramolecular H-bonds. Interactions between C9–H∙∙∙O5 [D∙∙∙A = 3.331(3) Å], C5–H∙∙∙O6 [D∙∙∙A = 3.529(3) Å], C19–H∙∙∙O3 [D∙∙∙A = 3.316(3) Å], C16–H∙∙∙O5 [D∙∙∙A = 3.315(3) Å], and C9–H∙∙∙O4 [D∙∙∙A = 3.313(3) Å] contribute to the formation of a stable structure (Figure 2a and Figure 2b).

**Scheme 4:**
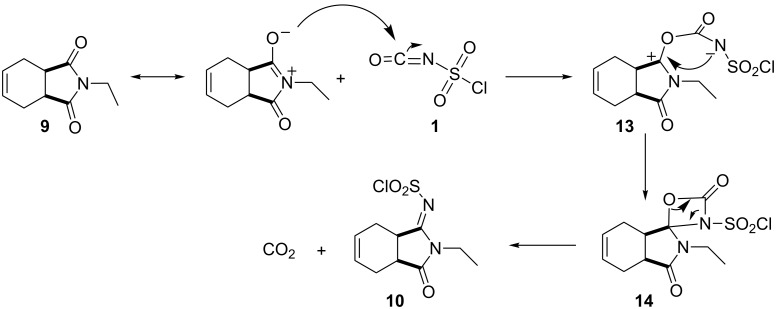
Mechanism for the formation of ylidenesulfamoyl chloride **10**.

Based on the structure of the product, we propose the reaction mechanism shown in Scheme 4. First, CSI reacts with the carbonyl carbon in the imide ring to form a four-membered urethane ring. Afterwards the imine is formed by the release of carbon dioxide from the molecule.

**Figure 3:**
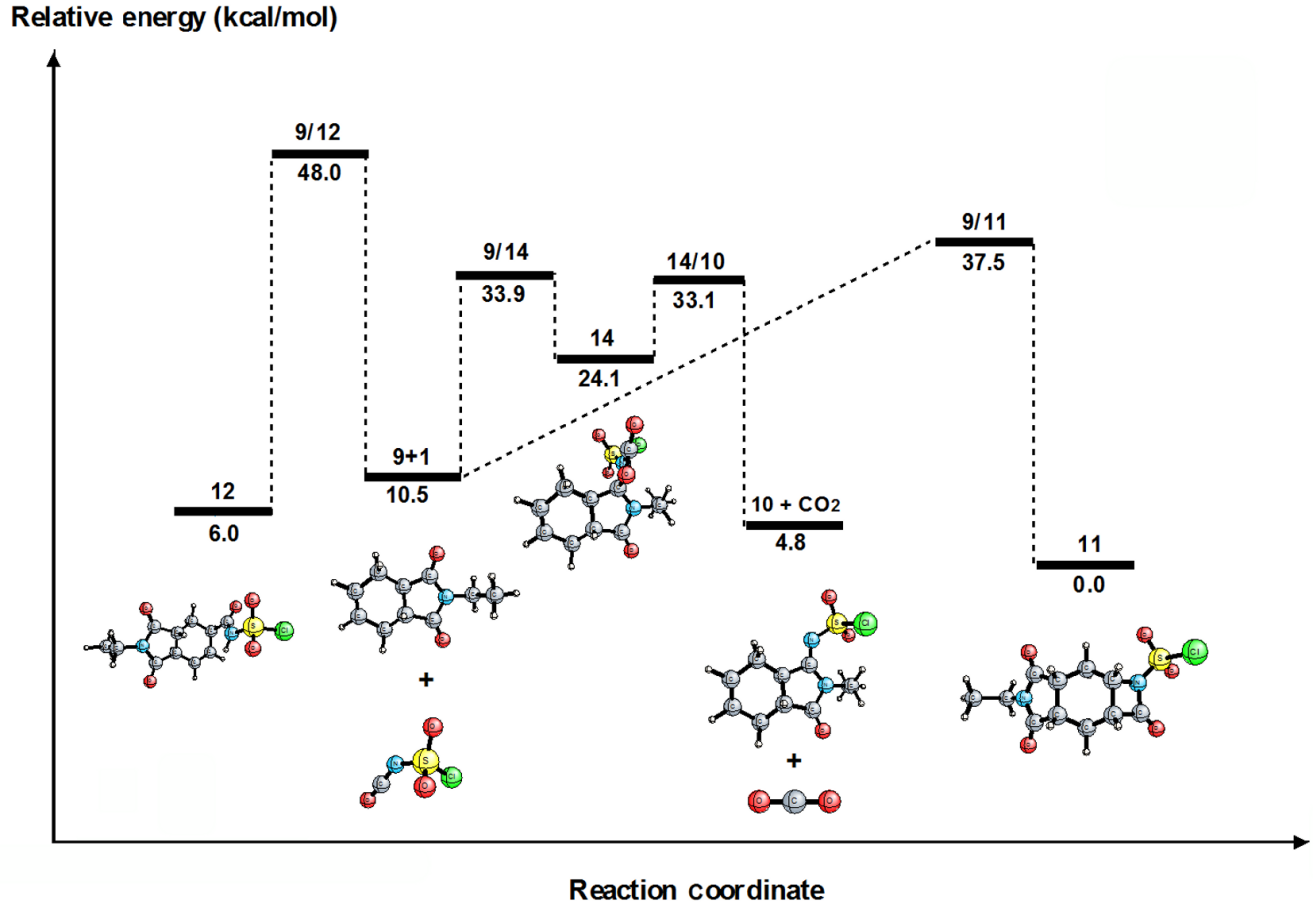
Relative energy profile of the reaction mechanism shown in Scheme 4.

We performed theoretical computations to better understand the reaction mechanism shown in Scheme 4 (Figure 3). For this purpose, we employed density functional theory (DFT) calculations and performed geometric optimizations using the B3LYP functional [19–22]. We computed the vibrational frequencies to characterize each stationary structure. In all the computations, we utilized Pople’s polarized triple-ζ split valence basis set with diffuse functions, 6-311++G(d,p) [23–25]. All the computations were performed using the Gaussian 09 program package [26]. The energies of all the structures are on the B3LYP/6-311G++(d,p) level, and the zero-point vibrational energy (ZPVE) corrections are all at the DFT level. Throughout this study, all the relative energies refer to the ZPVE-corrected energies. For the transition state (TS) between species A and B, we use the notation A/B throughout the article.

Figure 3 shows the relative energy profile for the reaction mechanism shown in Scheme 4. The rate-determining steps for the formation of **10**, **11**, and **12** are the transition states **9/14**, **9/11**, and **9/12**, respectively. The difference between the reaction barriers for the formations of **10** and **11** is 3.6 kcal/mol, whereas that for the formations of **10** and **12** is 14.1 kcal/mol. Hence, the formation of **10** is kinetically more favorable. This computational result is consistent with the experimental observations.

## Conclusion

For the first time, we have demonstrated the addition of chlorosulfonyl isocyanate to the system comprising both independent double bonds and imide functional groups. The mechanism for the addition of CSI to a carbonyl group is explained by theoretical computations. Supported by theoretical calculations, we determined the reaction mechanism of the addition product. Such an addition reaction is one of the unique examples that define the addition of CSI. Furthermore, the chemical transformation of chlorosulfonyl isocyanate to the related compounds is currently under investigation.

## Experimental

### General

All reagents and substrates were purchased from commercial sources and used without further purification. Solvents were purified and dried by standard procedures before use. ^1^H and ^13^C NMR spectra were recorded on Varian 400 and Bruker 400 spectrometers. Elemental analyses were performed on a Leco CHNS-932 instrument. The melting points were measured with Gallenkamp melting point devices. X-ray crystallography was performed using a Rigaku R-AXIS RAPID IP diffractometer. HRMS: electron-spray technique (M^+^/M^−^) from the solution in MeOH (Waters LCT PremierTM XE UPLC/MS TOF (Manchester, UK)). All the computations were performed using the Gaussian 09 program package. The energies of all the structures are on the B3LYP/6-311G++(d,p) level.

**((3a*S*,7a*R*,*E*)-2-Ethyl-3-oxo-2,3,3a,4,7,7a-hexahydro-1*H*-isoindol-1-ylidene)sulfamoyl chloride (10):** The synthesis of 2-ethyl-3-oxo-2,3,3a,4,7,7a-hexahydro-1*H*-isoindol-1-ylidene)sulfamoyl chloride started from (3a*R*,7aS)-2-ethyl-3a,4,7,7a-tetrahydro-1*H*-isoindole-1,3(2*H*)-dione (0.50 g, 2.79 mmol). The starting material was put into a round bottomed flask and N_2_ was passed throughout the flask. Chlorosulfonyl isocyanate (CSI, 0.73 mL, 8.37 mmol) was added and reaction mixture was stirred at 80 °C for 4 h. At the end of this time, it was cooled to rt and diluted with EtOAc. Unreacted CSI and solvent were removed in vacuo and the residue was dissolved in CH_2_Cl_2_. It was filtered via a column and concentrated in vacuo. The residue was crystallized from CH_2_Cl_2_/hexane to obtain ((3a*S*,7a*R*,*E*)-2-ethyl-3-oxo-2,3,3a,4,7,7a-hexahydro-1*H*-isoindol-1-ylidene)sulfamoyl chloride (**10**, 0.233 g, colorless crystalline solid, 30% yield). ^1^H NMR (400 MHz, CDCl_3_) δ 5.93–5.89 (m, 1H, A of AB system, H6), 5.86–5.81 (m, 1H B of AB system, H5), 4.09 (m, 1H, H7a) 3.68 (q, *J* = 7.2 Hz 2H, N*-*C*H_2_*-), 3.10 (m, 1H, H3a), 2.70–2.61 (m, 2H, 2 × H4), 2.47–2.40 (m, 1H, H7(axial)), 2.34–2.26 (m, 1H, H7(equatorial)), 1.17 (t, 3H, -*CH_3_*, *J* = 7.2 Hz.); ^13^C NMR (100 MHz, CDCl_3_) δ 179.7 (C1), 178.1 (C3), 127.6 (C6), 126.0 (C5), 39.5 (C7a), 37.9 (C3a), 36.5 (*N-CH_2_*-), 25.5 (C7), 22.4 (C4), 12.6 (-*C*H_3_); mp 68–70 °C; HRMS (APCI): [M + H]^+^ calcd for C_10_H_13_ClN_2_O_3_S, 276.7350; found, 277.0434.

## Supporting Information

File 1 Theoretical computations, experimental procedures, copies of ^1^H and ^13^C NMR spectra, X-ray diffraction and HRMS analysis.
