# Supplementary material for: An anomalous addition of chlorosulfonyl isocyanate to a carbonyl group: the synthesis of ((3aS,7aR,E)-2-ethyl-3-oxo-2,3,3a,4,7,7a-hexahydro-1H-isoindol-1-ylidene)sulfamoyl chloride
Source: Beilstein J Org Chem. 2019 Apr 16;15:931–6. doi: 10.3762/bjoc.15.89 (PMC6541324; doi:10.3762/bjoc.15.89)
Supplement: File 1 — Theoretical computations, experimental procedures, copies of 1H and 13C NMR spectra, X-ray diffraction and HRMS analysis. [file Beilstein_J_Org_Chem-15-931-s001.pdf]

BEILSTEIN JOURNAL OF ORGANIC CHEMISTRY

Supporting Information

for

An anomalous addition of chlorosulfonyl isocyanate to a carbonyl group: the synthesis of ((3a*S*,7a*R*,*E*)-2-ethyl-3-oxo-2,3,3a,4,7,7a-hexahydro-1*H*-isoindol-1-ylidene)sulfamoyl chloride

Aytekin Köse, Aslı Ünal, Ertan Şahin, Uğur Bozkaya and Yunus Kara

Beilstein J. Org. Chem. **2019,** *15,* 931–936. doi:10.3762/bjoc.15.89

Theoretical computations, experimental procedures, copies of ^{1}H and ^{13}C NMR spectra, X-ray diffraction and HRMS analysis

License and Terms: This is a supporting information file under the terms of the Creative Commons Attribution License (http://creativecommons.org/licenses/by/4.0). Please note that the reuse, redistribution and reproduction in particular requires that the authors and source are credited.
The license is subject to the *Beilstein Journal of Organic Chemistry* terms and conditions: (https://www.beilstein-journals.org/bjoc)

Table of contents

Geometries computed at the B3LYP/ 6311++G(d,p) ………………………….. S3

Experimental procedure and spectroscopic datas …………………………….. S14

Copies of 400 MHz ^{1}H NMR and 100 MHz ^{13}C NMR spectrum of ((3a*S*,7a*R,E*)-2-ethyl-3-oxo-2,3,3a,4,7,7a-hexahydro-1*H*-isoindol-1-ylidene)sulfamoyl chloride 10 in $CDCl_3$ ……………………………………………………………..… S15

Copy of HETCOR spectrum of compound 10 ……………………………..... S16

X-ray diffraction analysis for 10 …………………………………………...…. S17

HRMS analysis report of compound 10 …………………………………...… S18

Theoretical computations: Geometries Computed at the B3LYP/ 6311++G(d,p) Level

1

Number of imaginary frequencies: 0
Total Energy: -1176.978604 a.u.
ZPVE: 0.024184 a.u.

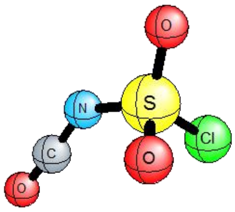

0 1			
S	0.51749700	-0.53733100	0.05442600
O	1.32015000	-1.34670000	-0.81960600
O	0.28384300	-0.85977300	1.44208600
Cl	1.28687100	1.41699100	-0.00969200
N	-0.95095300	-0.30214200	-0.72822000
C	-2.00141700	0.09821900	-0.23957900
O	-3.04044100	0.46074100	0.10614000

9

Number of imaginary frequencies: 0

Total Energy: -594.281333 a.u.

ZPVE: 0.217658 a.u.

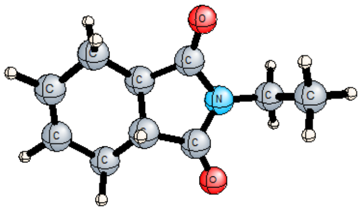

0 1

C	-2.01076600	1.51126400	-0.14935700
C	-3.23565700	0.64848100	0.09040400
C	-0.78146200	0.70692200	0.24664200
C	-0.83531900	-0.70633700	-0.34106200
C	-3.20572200	-0.67088200	0.31584100
C	-1.94995100	-1.52213300	0.29713600
C	0.62319000	1.17323100	-0.09584900
C	0.61386200	-1.15952600	-0.29449200
N	1.38687400	0.01003800	-0.27221800
C	2.84281000	0.01863000	-0.42632700
C	3.57940000	-0.02273800	0.91261300
O	1.05761100	2.29942300	-0.16103500
O	1.06243500	-2.28143300	-0.33468900
H	-2.08048000	2.43450900	0.43315900
H	-1.95726600	1.81988900	-1.20133600
H	-4.19496500	1.15838200	0.09305800
H	-0.78183800	0.60925400	1.34258900
H	-1.06062600	-0.60961700	-1.41363500
H	-4.14021400	-1.18981200	0.51012600
H	-2.12992600	-2.44583700	-0.26045100
H	-1.67923700	-1.83062700	1.31522100
H	3.10113200	-0.84905400	-1.03511300
H	3.09592000	0.92547200	-0.97825100
H	4.65909800	-0.01345400	0.74164500
H	3.32678900	0.84600600	1.52477300
H	3.33081900	-0.93077200	1.46624300

9/14

Number of imaginary frequencies: 1 (197i cm^{-1})

Total Energy: -1771.224271 a.u.

ZPVE: 0.243541 a.u.

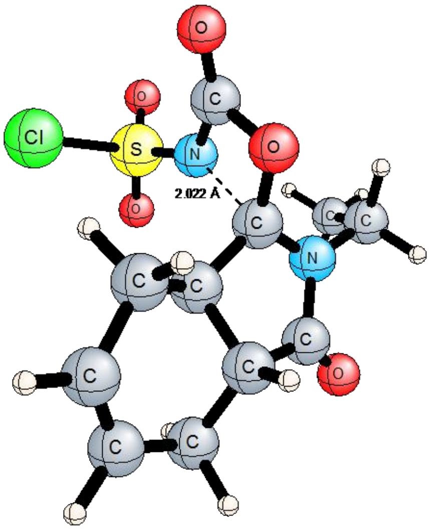

0 1

C	-1.17549300	-2.03469000	1.15183200
C	-2.09055100	-3.05114000	0.49458700
C	-1.20584600	-0.76929100	0.30671200
C	-2.97607000	-2.77479300	-0.46940600
C	-2.65078100	-0.37826100	-0.04745500
C	-3.25864500	-1.38603900	-1.01045300
C	-0.64022600	0.53773700	0.81821800
C	-2.53084700	1.08951400	-0.38691700
N	-1.37384900	1.55423100	0.34216800
C	-1.07821500	2.98953200	0.50983000
C	-0.33622000	3.57561000	-0.69077800
N	1.28876700	0.51062300	0.21276400
S	2.41015100	0.02256000	-0.87762900
O	1.73039100	0.01820200	-2.15909700
O	3.69945100	0.65319800	-0.69712600
Cl	2.69487000	-2.04870700	-0.37353600
C	1.44503100	0.48648600	1.56318100
O	2.31393200	0.34593400	2.35286300
O	0.05861400	0.70737700	1.96099600
H	-4.33699500	-1.23076800	-1.10610000
H	-2.84030100	-1.27061300	-2.01793100
H	-3.55476600	-3.58684300	-0.89892100
H	-1.98405800	-4.07778500	0.83117300
H	-0.16037000	-2.43716800	1.21308300
H	-1.49537400	-1.83579300	2.18221400
H	-0.69107700	-0.97412500	-0.63666700
H	-3.23920200	-0.39320200	0.88236900
H	-2.03939000	3.48553200	0.65138100
H	-0.50129000	3.08824500	1.42906700
H	-0.14535100	4.63550500	-0.50636600
H	0.62024200	3.07579400	-0.84651300
H	-0.93364100	3.48973600	-1.59945200
O	-3.23874600	1.81736300	-1.02086000

14

Number of imaginary frequencies: 0

Total Energy: -1771.241660 a.u.

ZPVE: 0.245334 a.u.

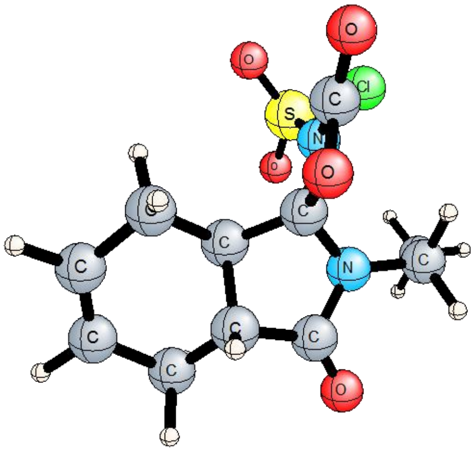

0 1

C	-1.80760600	-2.03250300	0.42720600
C	-3.12349000	-2.40937000	-0.22695800
C	-1.43522700	-0.62817400	-0.03176000
C	-3.94684600	-1.54660000	-0.83235900
C	-2.62843600	0.32619000	0.08388600
C	-3.71979800	-0.05041300	-0.90862700
C	-0.32577700	0.16342800	0.64943300
C	-1.97469100	1.69536500	0.03661300
N	-0.63691600	1.51197500	0.41178700
C	0.24567800	2.64682600	0.71803400
C	0.89160700	3.25307900	-0.52685400
N	1.09755500	-0.26302800	0.48035900
S	1.92450100	-0.85739600	-0.85276300
O	2.26284500	-2.24682100	-0.66641600
O	1.20642500	-0.36538200	-2.00795500
Cl	3.71652400	0.21702100	-0.69872000
C	1.13960100	-0.64367000	1.85001400
O	1.94044800	-1.15372900	2.55155200
O	-0.11803200	-0.17362500	2.09543100
H	-4.64689700	0.48461300	-0.68458300
H	-3.43915300	0.24293700	-1.92821000
H	-4.85283900	-1.92470600	-1.29696000
H	-3.38728200	-3.46243200	-0.20311700
H	-1.03836300	-2.76031800	0.14777300
H	-1.89713700	-2.07877500	1.51881100
H	-1.15344700	-0.66978800	-1.09012000
H	-3.03981500	0.23834700	1.09973500
H	-0.36599900	3.39341700	1.22830200
H	0.99980500	2.29865100	1.42571500
H	1.52913200	4.09261600	-0.23728600
H	1.50843200	2.52377400	-1.05511100
H	0.12764300	3.62496800	-1.21134500
O	-2.47063100	2.76932000	-0.20610700

14/10

Number of imaginary frequencies: 1 (359i cm^{-1})

Total Energy: -1771.224711 a.u.

ZPVE: 0.242780 a.u.

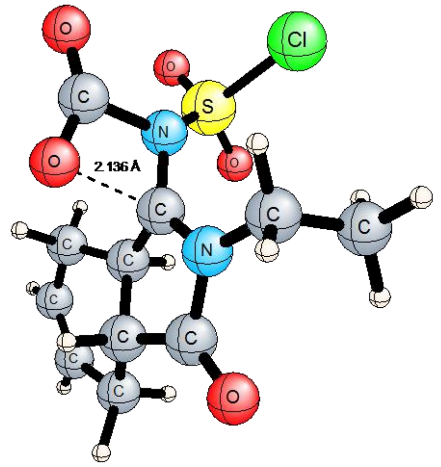

0 1

C	-1.85403500	-2.00349700	-0.03259900
C	-3.25545000	-2.21332000	-0.57268600
C	-1.46897600	-0.55168700	-0.28510200
C	-4.10615600	-1.23494200	-0.90116000
C	-2.59050200	0.40245300	0.14023700
C	-3.82389400	0.24572200	-0.73852100
C	-0.25938300	0.10174400	0.32039800
C	-1.88509700	1.72806800	0.26719200
N	-0.47840200	1.40651900	0.45176300
C	0.50326500	2.43621600	0.84130200
C	0.91508000	3.31816800	-0.33642800
N	1.01746200	-0.46256000	0.45642400
S	1.96424200	-0.79637600	-0.85798900
O	2.55545300	-2.10427300	-0.73519200
O	1.24218700	-0.36396100	-2.04505200
Cl	3.57490100	0.57079100	-0.62392800
C	0.92635400	-1.14131300	1.92474000
O	1.84349100	-1.77179100	2.33514200
O	-0.21075100	-0.75844000	2.27494900
H	-4.68552900	0.74711400	-0.28886200
H	-3.67154200	0.71825700	-1.71699200
H	-5.08057700	-1.49742400	-1.30182400
H	-3.56795200	-3.24507100	-0.70143600
H	-1.15941500	-2.68618400	-0.53112900
H	-1.81221700	-2.23440200	1.03617700
H	-1.30892900	-0.43194800	-1.36822600
H	-2.85354000	0.13318600	1.17450700
H	0.03079100	3.02874500	1.62627000
H	1.35739900	1.91051100	1.26537400
H	1.37535900	2.72741200	-1.12987600
H	0.05861400	3.85880600	-0.74052900
H	1.64883200	4.04949900	0.00993100
O	-2.29452000	2.85180000	0.28011100

10

Number of imaginary frequencies: 0

Total Energy: -1582.622915 a.u.

ZPVE: 0.231027 a.u.

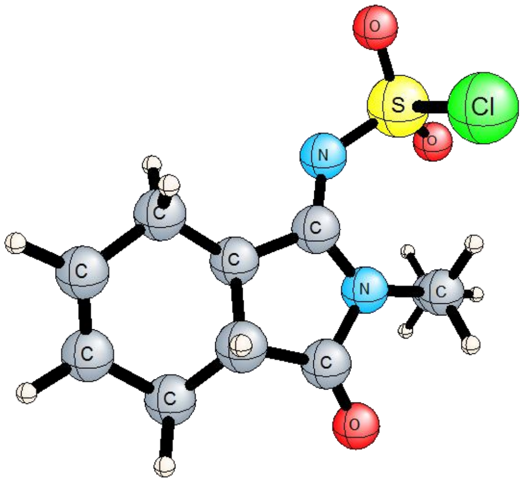

0 1

C	-1.95381800	-2.09169700	-0.15068800
C	-3.46761300	-2.18144300	-0.12902600
C	-1.57086200	-0.64447800	-0.41668300
C	-2.37285600	0.31152200	0.46506600
C	-4.29460700	-1.13180600	-0.05586600
C	-3.84589300	0.31027200	0.08414000
C	-0.14460700	-0.16178900	-0.23596300
C	-1.54594200	1.57076600	0.40491900
N	-0.20030600	1.16564600	0.10936700
C	0.85158700	2.20852800	0.10275800
C	0.99970900	2.86936000	-1.26528800
O	-1.86093800	2.71363500	0.59970600
N	0.80174000	-1.02421700	-0.43199200
S	2.44033300	-0.83548300	-0.38958800
O	3.01867100	-2.14196700	-0.59310800
O	2.90830300	0.30774800	-1.15733300
Cl	2.80624600	-0.37685700	1.69168700
H	-1.54587700	-2.75164800	-0.92057600
H	-1.53052200	-2.43974200	0.79958800
H	-3.89171500	-3.17897300	-0.19602600
H	-1.81465900	-0.41313300	-1.46448200
H	-2.28439200	-0.04146500	1.50329800
H	-5.36655100	-1.30442900	-0.08012100
H	-4.44519000	0.82027500	0.84370300
H	-4.01026600	0.85914800	-0.85177200
H	0.53400100	2.93877900	0.84712600
H	1.78545000	1.77190400	0.43686600
H	1.76216100	3.64966800	-1.20260800
H	1.32399800	2.14856500	-2.01671000
H	0.06315400	3.33460200	-1.57951100

9/11

Number of imaginary frequencies: 1 (385i cm^{-1})

Total Energy: -1771.216988 a.u.

ZPVE: 0.241966 a.u.

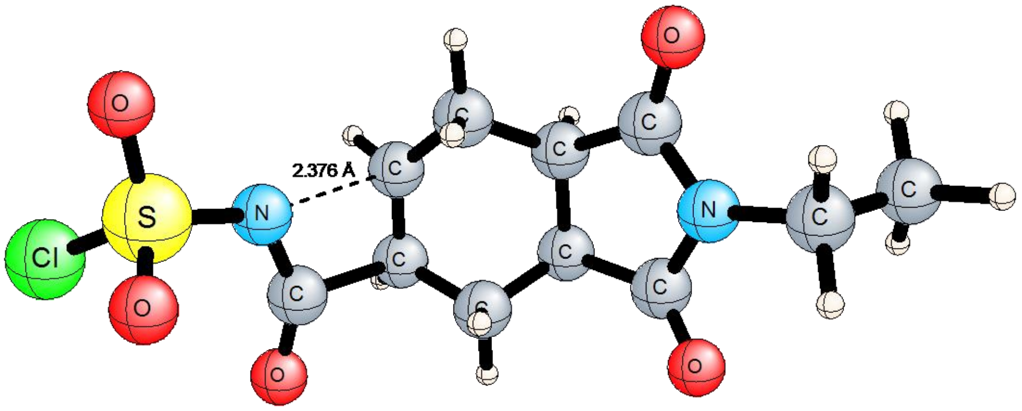

0 1			
C	-0.61543200	-0.94968100	1.29768500
C	0.36044100	0.07186500	1.74044500
C	-2.03959500	-0.31420500	1.32511800
C	-2.10516500	1.12558000	0.76916000
C	0.38179800	1.32389500	1.10757200
C	-0.77145000	1.60164600	0.14644100
C	-3.02618100	-1.11468100	0.47240300
C	-3.21902400	1.08412200	-0.27551400
N	-3.66974200	-0.23090200	-0.38619800
C	-4.71997700	-0.64492200	-1.32480600
C	-6.11659700	-0.56382700	-0.70999800
N	2.23010800	-0.16742400	0.29345800
C	1.87215500	1.09545800	0.10962600
O	2.20764500	2.06997300	-0.47612200
S	3.50875000	-0.77996600	-0.61104800
O	3.65610100	-2.15626000	-0.18620700
O	3.42859200	-0.42875300	-2.01569800
Cl	5.20306500	0.26847100	0.18462400
O	-3.22368500	-2.30457300	0.52527300
O	-3.63718800	2.01348400	-0.92219100
H	-0.57419300	-1.85976100	1.89255800
H	-0.36724500	-1.22820200	0.26628200
H	1.01728400	-0.12659900	2.57948200
H	-2.41014400	-0.34899900	2.35290000
H	-2.40362200	1.83623000	1.54431400
H	0.73075200	2.15431000	1.71912800
H	-0.60593000	1.07209700	-0.79790400
H	-0.82152100	2.66044800	-0.10370000
H	-4.63880800	0.00937900	-2.19350200
H	-4.48545500	-1.66647900	-1.62628500
H	-6.85968800	-0.88263900	-1.44495700
H	-6.19997000	-1.21790400	0.16067800
H	-6.35368400	0.45937600	-0.41089600

11

Number of imaginary frequencies: 0

Total Energy: -1771.281551 a.u.

ZPVE: 0.246803 a.u.

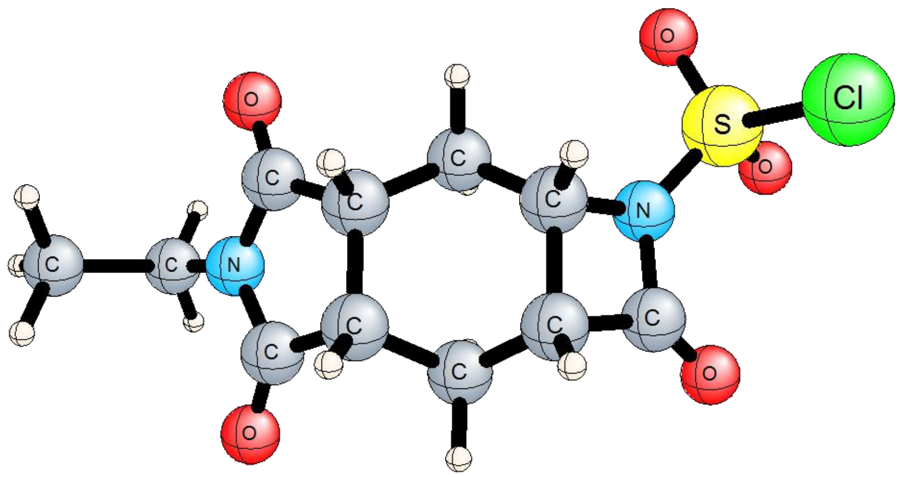

0 1

C	0.22202000	-0.92971400	-0.13595000
C	-0.89249500	-0.04693100	-0.68351400
C	1.54282600	-0.53998100	-0.83201200
C	1.86475100	0.97566900	-0.76387800
C	-0.57495600	1.48380400	-0.64915800
C	0.78972000	1.81383600	-0.04193600
C	2.74242200	-1.22277300	-0.17850400
C	3.21655500	1.05629600	-0.05837900
N	3.64311900	-0.23887100	0.22272100
C	4.91495500	-0.54048600	0.88821500
C	6.05749500	-0.75330400	-0.10451100
N	-2.01885100	0.23937800	0.25524600
C	-1.81884700	1.66194900	0.23097200
O	-2.42878500	2.55967700	0.71165200
S	-3.35593000	-0.70794200	0.57192800
O	-2.92093700	-2.08570900	0.51012700
O	-4.08966600	-0.14039900	1.67327100
Cl	-4.52632300	-0.38401700	-1.17628500
O	2.90032800	-2.40932900	-0.01598600
O	3.82525900	2.06114800	0.22486500
H	0.00969000	-1.98769900	-0.28969600
H	0.30563800	-0.77492600	0.94326800
H	-1.25743800	-0.39037300	-1.65395800
H	1.50877200	-0.88440400	-1.87023500
H	2.00731600	1.38552200	-1.76875100
H	-0.71565300	1.97165900	-1.61698700
H	0.79228800	1.58547700	1.02771200
H	1.01563500	2.87636200	-0.13405800
H	5.12809900	0.29842800	1.55172100
H	4.75082600	-1.43643500	1.48827400
H	6.97968700	-0.97489100	0.43839700
H	5.84586300	-1.59328000	-0.76949900
H	6.22550200	0.14308100	-0.70547500

9/12

Number of imaginary frequencies: 1 (1098i cm^{-1})

Total Energy: -1771.197760 a.u.

ZPVE: 0.239440 a.u.

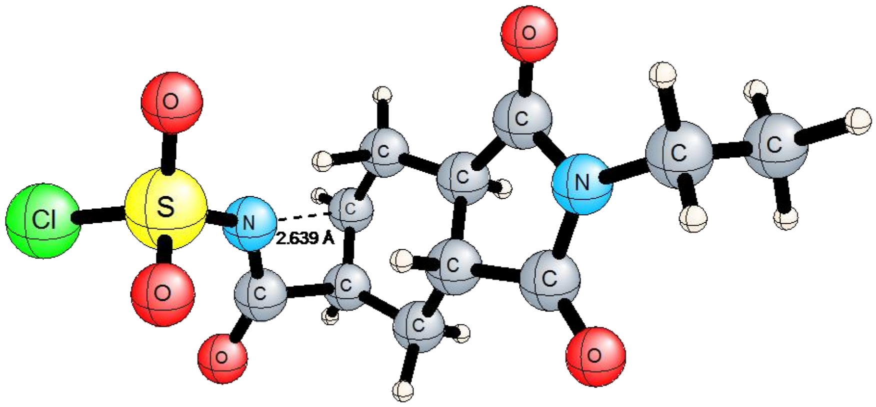

0 1

C	0.34561000	0.72208200	-1.99347000
C	-0.41702500	1.88486700	-1.75968500
C	1.24016500	0.88376400	0.37157600
C	-0.59921100	2.37470300	-0.41697400
C	0.59900600	2.25099300	0.56553700
C	1.56988800	0.61308200	-1.09719000
C	2.45049800	-0.62668000	-1.01803500
C	2.51851400	0.44183300	1.06756700
N	3.06595100	-0.55371000	0.23779300
C	4.17209100	-1.42653500	0.64590800
C	5.53860400	-0.87959500	0.23487400
C	-1.84950400	1.35616000	0.19633300
N	-1.73181100	0.12814200	-0.29356800
S	-2.53192000	-1.17628300	0.32788100
O	-2.17922800	-2.30456600	-0.50875700
O	-2.42265900	-1.24035400	1.77091900
Cl	-4.55945600	-0.71255100	-0.11137000
O	2.66225700	-1.46139400	-1.86218200
O	2.96756500	0.78336300	2.13258600
O	-2.58136700	1.91056000	0.97318000
H	0.41671800	0.34486900	-3.00985000
H	-1.10852200	2.23115600	-2.52508300
H	0.51415300	0.12553100	0.68732600
H	-1.08737200	3.34439300	-0.37139100
H	0.24538100	2.39196200	1.58791200
H	1.30948500	3.05652700	0.35574300
H	2.26997800	1.39167400	-1.43664900
H	4.10616200	-1.53065400	1.72963600
H	3.98863900	-2.40003900	0.18867900
H	6.32357700	-1.56486700	0.56444700
H	5.61166700	-0.78097400	-0.85065900
H	5.72383700	0.09397200	0.69376200
H	-0.61556600	0.14040400	-1.40087100

12

Number of imaginary frequencies: 0

Total Energy: -1771.270401 a.u.

ZPVE: 0.245267 a.u.

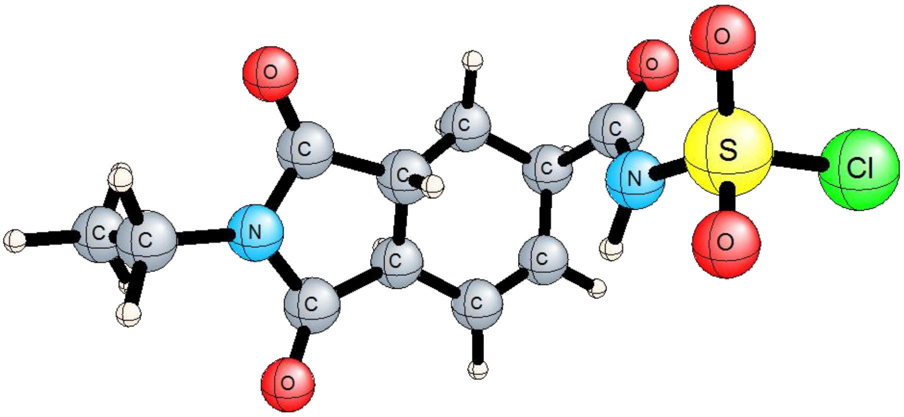

0 1

C	0.94444800	1.95309000	-1.11232000
C	-0.20653000	2.17499600	-0.46605900
C	1.47833400	0.23091400	0.61584100
C	-0.52878700	1.68703000	0.93957700
C	0.65475600	0.95652100	1.66504000
C	2.03866800	1.21628000	-0.40880600
C	3.09570400	0.38976100	-1.12953400
C	2.69410600	-0.62362700	0.94588500
N	3.50976400	-0.57174100	-0.19739000
C	4.68053900	-1.43236800	-0.39315200
C	5.97246800	-0.79592100	0.11868800
C	-1.78248400	0.79454200	0.99769400
N	-1.98268800	0.01100000	-0.15577800
S	-3.25775300	-1.05724800	-0.41706600
O	-3.05382000	-1.54153800	-1.76496600
O	-3.42572300	-1.92094900	0.72038100
Cl	-4.92457500	0.22667500	-0.47401800
O	3.55750300	0.54052400	-2.23409400
O	2.91987800	-1.28487400	1.92893100
O	-2.50331400	0.73182900	1.95780200
H	1.12162000	2.35583800	-2.10415800
H	-0.98188500	2.76347000	-0.95054000
H	0.81681900	-0.46291300	0.07692100
H	-0.81584800	2.54824900	1.54980100
H	0.26771700	0.28224300	2.43059400
H	1.26999300	1.70242100	2.17558400
H	2.64039900	1.96305700	0.13601300
H	4.47650700	-2.36750500	0.13021100
H	4.74469000	-1.63544800	-1.46323400
H	6.81114500	-1.47595700	-0.05078600
H	6.18437900	0.13822100	-0.40669200
H	5.91031800	-0.59422100	1.19010300
H	-1.49707600	0.26296900	-1.01184900

CO_2

Number of imaginary frequencies: 0

Total Energy: -188.615854 a.u.

ZPVE: 0.011688 a.u.

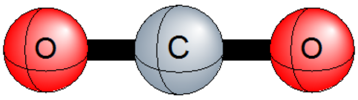

0 1			
C	-1.19636958	0.31353135	0.00000000
O	0.06203042	0.31353135	0.00000000
O	-2.45476958	0.31353135	0.00000000

Experimental procedure:

General

All reagents and substrates were purchased from commercial sources and used without further purification. Solvents were purified and dried by standard procedures before use. ^{1}H and ^{13}C NMR spectra were recorded on Varian 400 and Bruker 400 spectrometers. Elemental analyses were performed on a Leco CHNS-932 instrument. The melting points were measured with Gallenkamp melting point devices. X-ray crystallography was performed using a Rigaku R-AXIS RAPID IP diffractometer. HRMS: electron spray technique (M+/M−) from the solution in MeOH (Waters LCT PremierTM XE UPLC/MS TOF (Manchester, UK)). All the computations were performed using the Gaussian 09 program package. The energies of all the structures are on the B3LYP/6-311G++(d,p) level.

((3a*S*,7a*R*,*E*)-2-Ethyl-3-oxo-2,3,3a,4,7,7a-hexahydro-1*H*-isoindol-1-ylidene)sulfamoyl chloride (10): The synthesis of 2-ethyl-3-oxo-2,3,3a,4,7,7a-hexahydro-1*H*-isoindol-1-ylidene)sulfamoyl chloride started from (3a*R*,7aS)-2-ethyl-3a,4,7,7a-tetrahydro-1*H*-isoindole-1,3(2*H*)-dione (0.50 g, 2.79 mmol). The starting material was put into a round bottomed flask and N_2 was passed throughout the flask. Chlorosulfonyl isocyanate (CSI, 0.73 mL, 8.37 mmol) was added and reaction mixture was stirred at 80 °C for 4 h. At the end of this time, it was cooled to rt and diluted with EtOAc. Unreacted CSI and solvent were removed in vacuo and the residue was dissolved in CH_2Cl_2. It was filtered via a column and concentrated in vacuo. It was crystallized from CH_2Cl_2/hexane to obtain ((3a*S*,7a*R*,*E*)-2-ethyl-3-oxo-2,3,3a,4,7,7a-hexahydro-1*H*-isoindol-1-ylidene)sulfamoyl chloride (**10**, 0.233 g, colorless crystalline solid, 30% yield). ^{1}H NMR (400 MHz, $CDCl_3$) δ 5.93–5.89 (m, 1H, A of AB system, H6), 5.86–5.81 (m, 1H B of AB system, H5), 4.09 (m, 1H, H7a) 3.68 (q, J = 7.2 Hz 2H, N-CH_2-), 3.10 (m, 1H, H3a), 2.70–2.61 (m, 2H, 2 × H4), 2.47–2.40 (m, 1H, H7(axial)), 2.34–2.26 (m, 1H, H7(equatorial)), 1.17 (t, 3H, -CH_3, J = 7.2 Hz.); ^{13}C NMR (100 MHz, $CDCl_3$) δ 179.7 (C1), 178.1 (C3), 127.6 (C6), 126.0 (C5), 39.5 (C7a), 37.9 (C3a), 36.5 (*N*-CH_2-), 25.5 (C7), 22.4 (C4), 12.6 (-*C*H_3); mp 68–70°C; HRMS (APCI): $[M + H]^+$ calcd for $C_{10}H_{13}ClN_2O_3S$, 276.7350; found, 277.0434.

400 MHz ^{1}H NMR spectrum of ((3a*S*,7a*R*,*E*)-2-ethyl-3-oxo-2,3,3a,4,7,7a-hexahydro-1*H*-isoindol-1-ylidene)sulfamoyl chloride (**10**) in $CDCl_3$

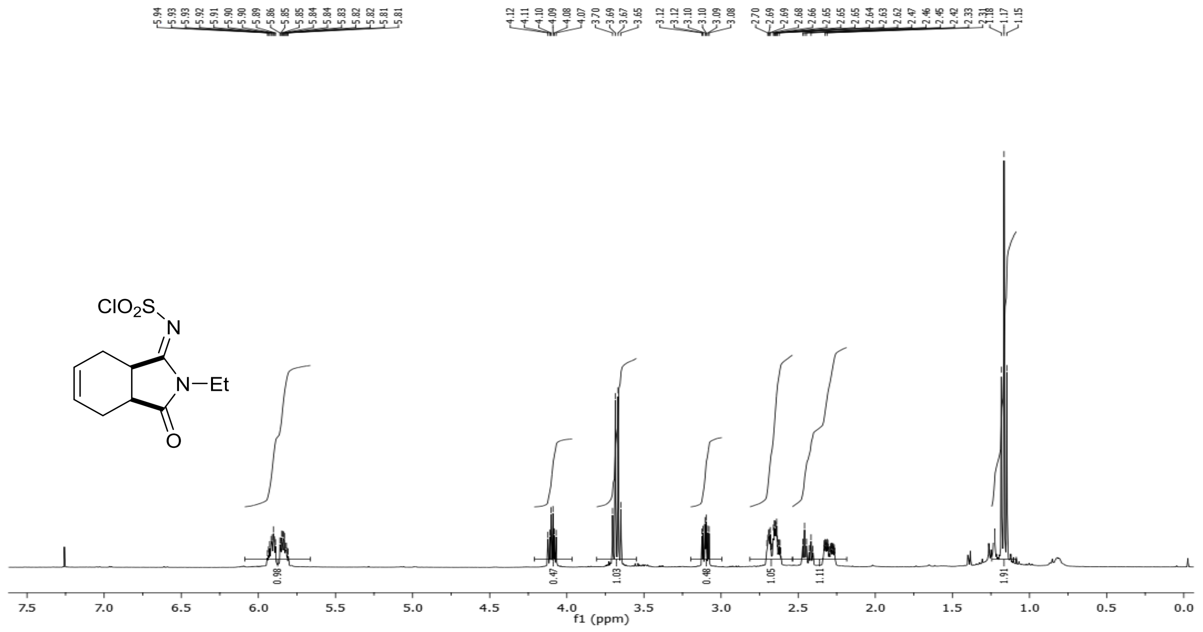

100 MHz ^{13}C NMR spectrum of ((3a*S*,7a*R*,*E*)-2-ethyl-3-oxo-2,3,3a,4,7,7a-hexahydro-1*H*-isoindol-1-ylidene)sulfamoyl chloride (**10**) in $CDCl_3$

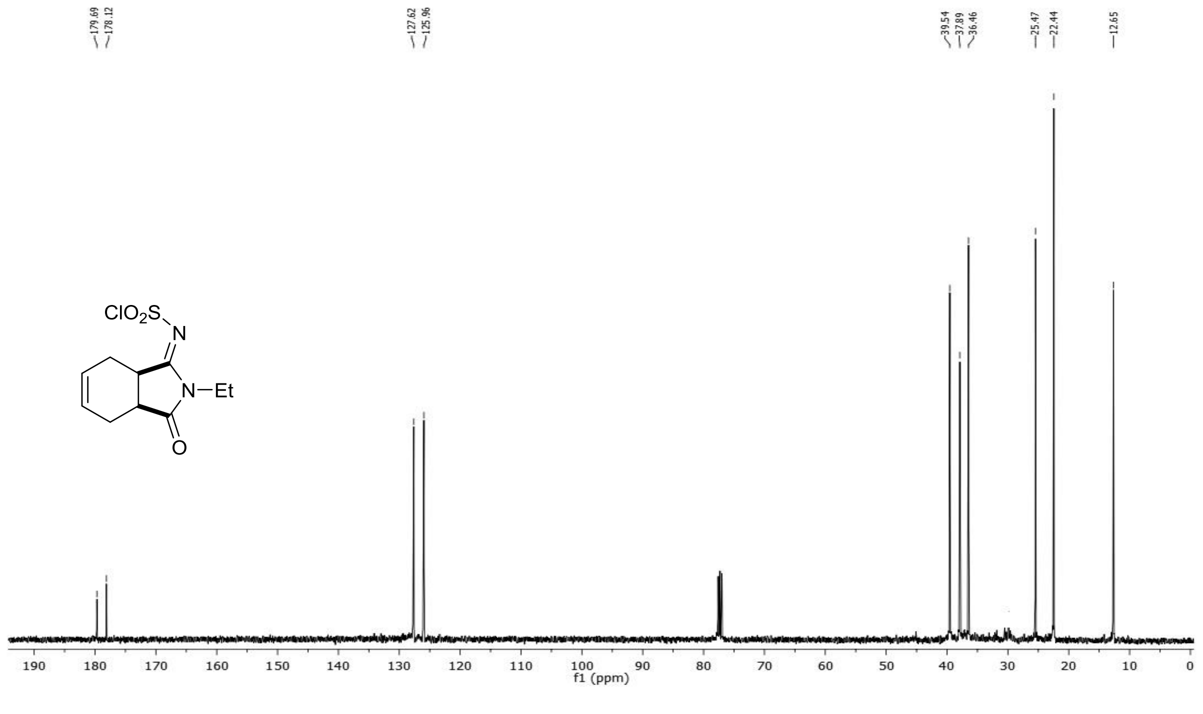

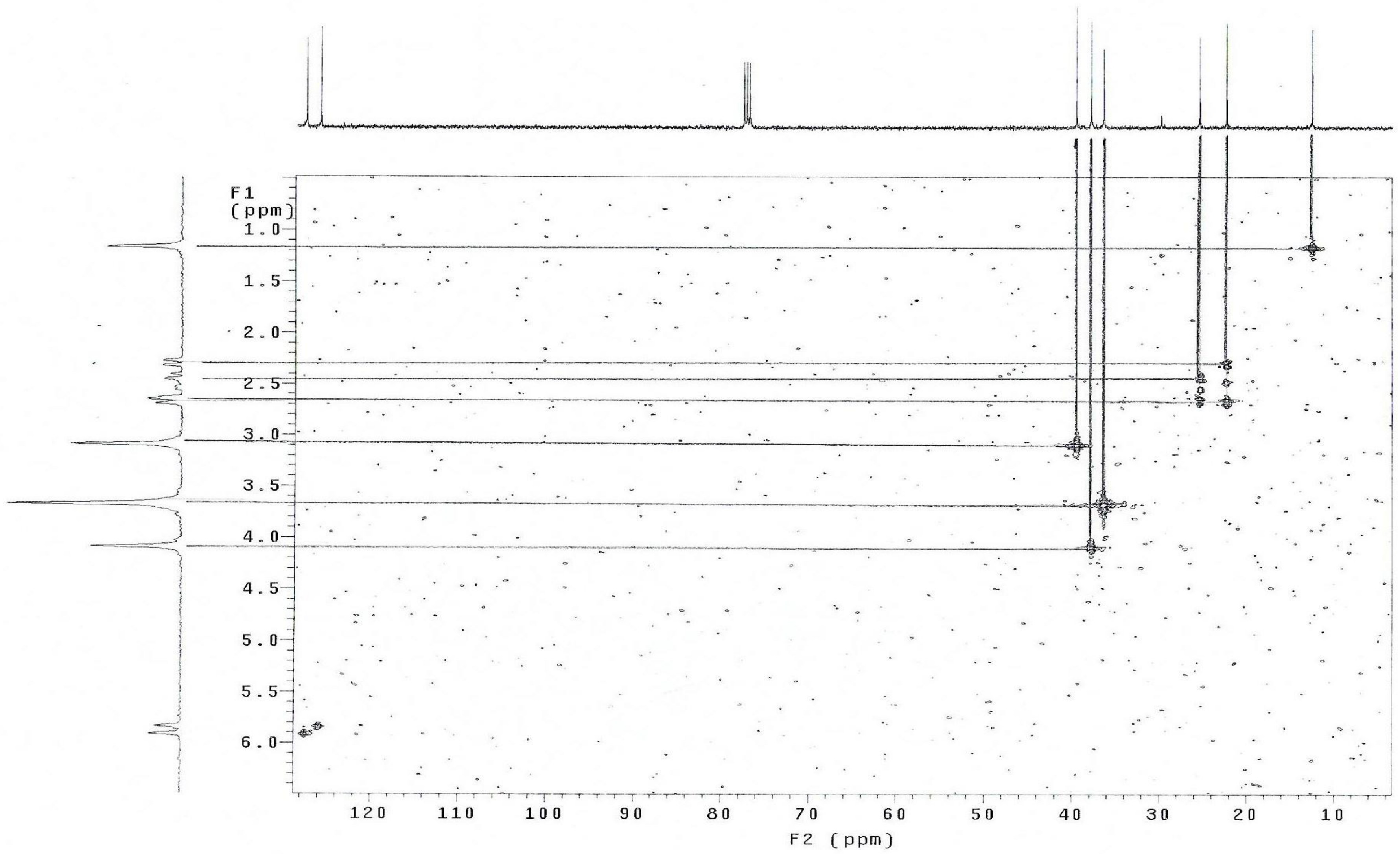

HETCOR spectrum of compound **10**

X-ray diffraction analysis:

For the crystal structure determination, a suitable single-crystal of compound **10** was used for data collection on a four-circle Rigaku R-AXIS RAPID-S diffractometer (equipped with a two-dimensional area IP detector). Graphite-monochromated Mo-K_{α} radiation (λ = 0.71073 Å) and oscillation scans technique with Δw = 5º for one image were used for data collection. The lattice parameters were determined by the least-squares methods on the basis of all reflections with $F^2 > 2\sigma(F^2)$. Integration of the intensities, correction for Lorentz and polarization effects and cell refinement was performed using CrystalClear (Rigaku/MSC Inc.,2005) software. The structures were solved by direct methods using SHELXS-97, which allowed location of most of the heaviest atoms, with the remaining non-hydrogen atoms being located from difference Fourier maps calculated from successive full-matrix least squares refinement cycles on F^2 using SHELXL-97. All non-hydrogen atoms were refined using anisotropic displacement parameters. Hydrogens attached to carbons were located at their geometric positions using appropriate HFIX instructions in SHELXL. The final difference Fourier maps showed no peaks of chemical significance. Crystal data for **10**: $C_{10}H_{13}N_2O_3SCl$, crystal system, space group: monoclinic, *C*2/*c*; (no:15); unit cell dimensions: *a* = 25.777(4), *b* = 12.9001(19), *c* = 19.295(3)Å, α = 90, β = 126.950(4), γ = 90º; volume: 5127.6(15) $Å^3$; Z = 8; calculated density: 1.434g/cm^3; absorption coefficient: 0.459 mm^{-1} ; *F*(000)= 2304; θ-range for data collection 2.6–25.2º ; refinement method: full matrix least-square on F^2; data/parameters: 3194/309; goodness-of-fit on F^2: 1.108; final *R*-indices [I > 2σ(I)]: R_1 = 0.050, wR_2 = 0.123; largest diff. peak and hole: 0.230 and -0.380 e $Å^{-3}$.

Crystallographic data for the structure **10** reported in this paper have been deposited with the Cambridge Crystallographic Data Center as supplementary publication No. CCDC-1852080 Copies of these data can be obtained free of charge on application to CCDC, 12 Union Road, Cambridge CB2 1EZ, UK; FAX: (+44) 1223 336033, or online via www.ccdc.cam.ac.uk/data_request/cif, or by emailing data_request@ccdc.cam.ac.uk.

HRMS Analysis:

Qualitative Analysis Report

**Data Filename**	A1.d	**Sample Name**	A1
**Sample Type**	Sample	**Position**	P1-F6
**Instrument Name**	Instrument 1	**User Name**	
**Acq Method**	MS_APCI_POS.m	**Acquired Time**	9/19/2017 3:07:39 PM
**IRM Calibration Status**	Success	**DA Method**	QualDAMethod.m
**Comment**			

**Sample Group**		**Info.**	
**Stream Name**	LC 1	**Acquisition SW Version**	6200 series TOF/6500 series Q-TOF B.06.01 (B6157)

User Chromatograms

Fragmentor Voltage 60 **Collision Energy** 0 **Ionization Mode** APCI

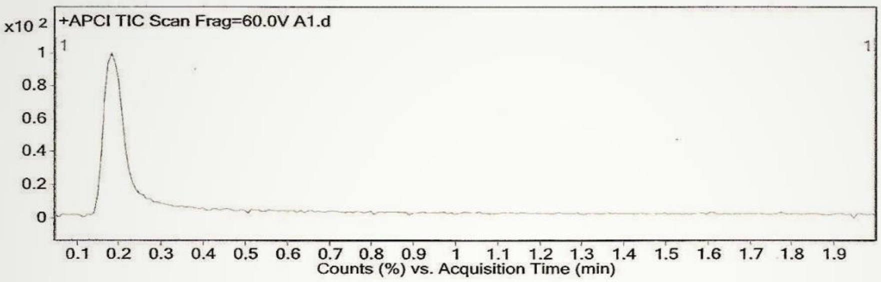

User Spectra

Fragmentor Voltage	Collision Energy	Ionization Mode
60	0	APCI

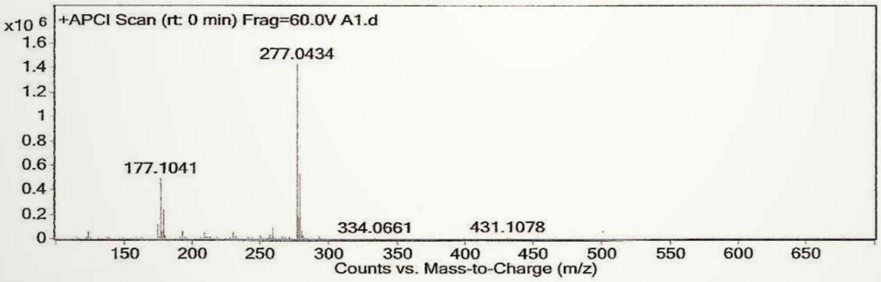

Peak List

m/z	z	Abund
123.9727		13682.93
124.0882	1	54497.11
138.0674		11855.25
159.0938		8975.93
175.0886	1	116403.69
176.0932	1	17237.7
177.1041	1	492745.06
178.1071	1	59354.06
179.1197	1	235074

Agilent Technologies Page 1 of 2 Printed at: 11:15 AM on: 9/26/2017

Qualitative Analysis Report

180.1215	1	27642.4
191.0849		12715.07
193.0995	1	57779.13
195.114		15777.61
206.0951		8865.7
209.0943		43648.95
210.0975		15224.85
211.1085		13322.29
212.1101		11430.78
213.0829		14795.38
218.1316		11955.94
228.115		9640.79
230.1309	1	50960.41
232.1467		23517.46
241.0679		15462.87
244.136		10310.46
250.1215	1	24198.95
257.1429	1	31865.28
259.0781	1	87638.07
259.1564	1	18714.52
260.0817	1	9841.14
266.1107		14864.14
268.1213	1	10422.17
271.1587		10438.76
277.0434	1	1430916.5
278.0476	1	173090.41
279.0415	1	528349.19
280.0446	1	63505.08
281.0395	1	23868.87
293.0397		15895.32
431.1078		11682.17

--- End Of Report ---

Agilent Technologies Page 2 of 2 Printed at: 11:15 AM on: 9/26/2017

HRMS analysis report of compound **10**
